# Interrupción de servicios de salud para embarazadas, recién nacidos, niños y niñas, adolescentes y mujeres durante la pandemia de COVID-19: proyecto ISLAC 2020

**DOI:** 10.26633/RPSP.2021.140

**Published:** 2021-11-03

**Authors:** Pablo Villalobos Dintrans, Matilde Maddaleno, Yamileth Granizo Román, Paula Valenzuela Delpiano, Arachu Castro, Carina Vance, Claudio A. Castillo

**Affiliations:** 1 Universidad de Santiago de Chile Santiago Chile Universidad de Santiago de Chile, Santiago, Chile.; 2 Escuela de Salud Pública y Medicina Tropical Universidad de Tulane Nueva Orleans Estados Unidos de América Escuela de Salud Pública y Medicina Tropical, Universidad de Tulane, Nueva Orleans, Estados Unidos de América

**Keywords:** Servicios de salud, infecciones por coronavirus, niños, adolescentes, mujeres, mujeres embarazadas, América Latina, región del Caribe, Health services, coronavirus infections, child, adolescent, women, pregnant women, Latin America, Caribbean region, Serviços de saúde, infecções por coronavirus, criança, adolescente, mulheres, gestantes, América Latina, região do Caribe

## Abstract

**Objetivos.:**

Describir la percepción de actores clave sobre la interrupción de los servicios de salud para poblaciones no priorizadas por la pandemia —embarazadas, recién nacidos, niños y niñas, adolescentes y mujeres— en países de América Latina y el Caribe (ALC) durante la primera etapa de la pandemia de COVID-19.

**Métodos.:**

Estudio transversal. Se aplicó una encuesta a actores relevantes de 19 países de ALC entre julio y septiembre del 2020, con 35 preguntas sobre su percepción personal del estado de los servicios sociales y de salud en su país antes y durante la pandemia, así como una proyección para después de ella.

**Resultados.:**

En las 691 respuestas, predominó la percepción de que la cobertura de servicios analizados era alta antes de la pandemia, aunque su calidad se apreció menor. Se percibió una reducción de la cobertura y la calidad de los servicios a adolescentes y mujeres. La mayoría estimó que todos los servicios seguirían con una menor cobertura tanto a los 3 como a los 12 meses (53,1% y 41,3%, respectivamente). Garantizar la cobertura y el acceso a los servicios de salud es el principal desafío político con vista al futuro, seguido del financiamiento de iniciativas para mujeres, niños, niñas y adolescentes, y la protección y promoción contra la violencia.

**Conclusiones.:**

Aunque la pandemia ha golpeado a todos los países, la afectación en la provisión de servicios para las poblaciones analizadas es heterogénea entre países y tipos de servicio. Se requiere invertir en sistemas de información nacionales que permitan monitorear los distintos servicios e identificar las poblaciones que no se han priorizado.

## Abstract (en)

**Objective.:**

Describe the perceptions of key actors regarding the disruption of health services for populations that ceased to be prioritized because of the COVID-19 pandemic—pregnant women, newborn, children, adolescents, and women—in countries of Latin America and the Caribbean (LAC) during the first stage of the pandemic.

**Methods.:**

In this cross-sectional study, a 35-question survey was administered to key actors in 19 LAC countries between July and September 2020. The respondents were asked for their personal perceptions regarding the situation of social and health services in their country before and during the pandemic. They were also asked for a projection of the situation during the post-pandemic period.

**Results.:**

In the 691 responses received, the main perception was that coverage in the services analyzed had been high before the pandemic, although their quality was not as highly rated. Both the coverage and quality of services were thought to have declined for adolescents and women. The majority of respondents predicted that all services will continue to function at lower than usual coverage levels for another three months (53.1%) and another 12 months (41.3%). Guaranteeing coverage and access to health services was considered the main policy challenge going forward. The next most needed initiatives noted were financing for actions to support women, children, and adolescents, and protection against violence and promotion of measures to combat it.

**Conclusions.:**

Although the pandemic has struck all countries, its effect on the delivery of services in the populations analyzed differs from country to country and according to the types of service. It is essential to invest in national information systems that will make it possible to monitor the different services and identify the populations that need to be prioritized.

## RESUMO

**Objetivos.:**

Descrever a percepção de atores-chave na interrupção dos serviços de saúde para populações não priorizadas na pandemia — grávidas, recém-nascidos, crianças, adolescentes e mulheres — em países da América Latina e do Caribe (ALC) durante a primeira fase da pandemia de COVID-19.

**Métodos.:**

Estudo transversal. Foi realizada uma pesquisa com atores relevantes de 19 países da ALC entre julho e setembro de 2020, com 35 perguntas sobre a percepção pessoal do estado dos serviços sociais e de saúde em seus países antes e durante a pandemia, bem como uma projeção para depois dela.

**Resultados.:**

Nas 691 respostas, predominou a percepção de que a cobertura dos serviços analisados era alta antes da pandemia, embora a qualidade fosse vista como mais baixa. Notou-se uma redução na cobertura e na qualidade dos serviços a adolescentes e mulheres. A maioria estimou que todos os serviços seguiriam com uma menor cobertura tanto em 3 como em 12 meses (53,1% e 41,3%, respectivamente). Garantir a cobertura e o acesso aos serviços de saúde é o principal desafio político para o futuro, seguido do financiamento de iniciativas para mulheres, crianças e adolescentes, e da proteção e promoção contra a violência.

**Conclusões.:**

Embora a pandemia tenha afetado todos os países, o abalo na provisão de serviços para as populações analisadas é heterogêneo entre os países e os tipos de serviço. É preciso investir em sistemas de informação nacionais que permitam monitorar os distintos serviços e identificar as populações que não foram priorizadas.

Desde su aparición a finales del 2019, la pandemia de COVID-19, causada por el coronavirus SARS-CoV-2, ha afectado negativamente a todo el mundo. Para agosto del 2021, más de 212 millones de personas habían contraído el virus y más de 4,4 millones habían muerto. La Región de las Américas —particularmente América Latina y el Caribe— ha sido de las más afectadas, con más de 82 millones de casos confirmados y más de 2 millones de muertes (1). Si bien esta situación ha requerido respuestas rápidas para abordar la crisis, estas pueden haber tenido consecuencias negativas en otras dimensiones, como el aumento de la incidencia y la gravedad de otros trastornos de salud por falta de acceso al tratamiento oportuno y adecuado (2, 3). Varios autores han alertado sobre el peligro de discontinuar los servicios esenciales de salud debido a su efecto a largo plazo (4, 5), y sobre la necesidad de adoptar un enfoque de salud integral en la toma de decisiones de políticas públicas (6–8).

Por ello, es importante analizar el efecto que las medidas de respuesta a la pandemia podrían provocar en poblaciones supuestamente menos afectadas por la COVID-19, en particular, niños, niñas y adolescentes (NNA), y mujeres. Dado que estos grupos —principalmente el de NNA— se identificaron como de bajo riesgo para la COVID-19 durante el 2020, existe la hipótesis de que su acceso a los servicios de salud se vio afectado y sus necesidades “invisibilizadas” durante esta etapa de la pandemia (5, 9), lo que puede haber conllevado a un retroceso en sus indicadores de salud y en el financiamiento a mediano y largo plazos de servicios esenciales de salud (4, 5, 10).

El proyecto “COVID-19 en América Latina y el Caribe: Impacto en los Servicios de Salud de la Mujer, la Niñez y la Adolescencia” (ISLAC) —desarrollado en colaboración por la Universidad de Tulane, Nueva Orleans, Estados Unidos de América, y la Universidad de Santiago de Chile, Santiago, Chile— tiene por objetivo identificar el impacto indirecto de la pandemia de COVID-19 en la salud de embarazadas, recién nacidos, niños y niñas, adolescentes y mujeres en 25 países de América Latina y el Caribe. Su fin es contribuir a adoptar políticas públicas mejor informadas y a asignar más adecuadamente los recursos en esta subregión (11). Esta línea de trabajo responde al Compromiso de Santiago, adoptado en el 2020 por los Estados Miembros de la Comisión Económica para América Latina y el Caribe (CEPAL), que insta a los gobiernos a abordar las causas estructurales de la desigualdad de género y promover políticas para responder a la pandemia en el corto, mediano y largo plazos (12).

El objetivo de este artículo es describir la percepción de actores clave sobre el efecto de la pandemia en la interrupción de los servicios y la atención de embarazadas, recién nacidos, niños y niñas, adolescentes y mujeres en países de América Latina y el Caribe durante la primera etapa de la pandemia de COVID-19.

## MATERIALES Y MÉTODOS

Se realizó un estudio transversal entre julio y septiembre del 2020 mediante una encuesta en línea basada en la herramienta de encuestas de Google (13). Se elaboró un cuestionario en español, francés, inglés y portugués que —además de recabar características sociodemográficas de los participantes— incluía 35 preguntas de selección múltiple mediante escalas Likert, sobre su percepción personal acerca del estado de los servicios sociales y de salud en cada país antes y durante la pandemia, así como una proyección para los siguientes meses. En la encuesta se solicitó considerar la cobertura, la calidad y la continuidad de los servicios de salud, la evaluación de las respuestas institucionales a la pandemia y la priorización de cinco grupos de la población: embarazadas, recién nacidos, niños y niñas, adolescentes y mujeres. En la encuesta también se hicieron preguntas específicas sobre la cobertura de salud para poblaciones vulnerables (migrantes, indígenas y afrodescendientes).

Se solicitó a los encuestados manifestar su percepción sobre las respuestas institucionales a la pandemia en sus países en los diferentes niveles de gobierno (central, federal o subnacional, y local), sectores (salud, educación, de otros organismos públicos y del sector privado) y tipo de institución (academia, organizaciones sin fines de lucro, y organismos y organizaciones internacionales). Las preguntas específicas sobre la respuesta dada por el sector salud se evaluaron también por nivel de atención (primario, secundario y terciario) y tipo de atención (promoción, prevención, cuidados, urgencia y rehabilitación). Finalmente, se solicitaba hacer una proyección sobre la evolución de los servicios en el corto y mediano plazos (3 y 12 meses, respectivamente) después de la pandemia y los desafíos a futuro. La validación de la encuesta la realizaron expertos y la aprobaron los comités de ética de las dos universidades patrocinadoras del proyecto.

Se invitó a participar, mediante correos electrónicos, a decisores e implementadores de programas y políticas sociales y de salud, tanto en el ámbito nacional como local, de 25 países de América Latina y el Caribe. La selección la realizó el equipo investigador con expertos regionales en el tema. Para conseguir una mayor cantidad de respuestas, se enviaron invitaciones por mensajería instantánea y redes sociales, en las que se solicitaba difundir la convocatoria entre decisores e implementadores de programas y políticas sociales y de salud, siguiendo una estrategia de *snowball sampling*, similar a lo utilizado por otros estudios de percepción en salud (14).

El análisis se realizó a partir del porcentaje de respuestas recibidas de cada país, agrupadas en cinco categorías: muy baja/muy mala, baja/mala, media/aceptable, alta/buena y muy alta/muy buena, y en dos niveles de agregación: por país y por el conjunto de países. Los datos también se agregaron por pregunta a partir del promedio simple de las respuestas por países y por agrupación de preguntas en temas, empleando para ello el promedio simple de las respuestas a preguntas relacionadas. Por ejemplo, el resultado de la percepción sobre “cobertura de servicios de salud” se obtuvo como el promedio de las cinco preguntas sobre cobertura (para embarazadas, recién nacidos, niños y niñas, adolescentes y mujeres)^1^.

Para el análisis de la cobertura antes y después de la pandemia, se construyó un índice de percepción para cada grupo de población estudiado en cada país, a partir del porcentaje correspondiente a cada categoría de respuesta. Este índice se obtuvo para cada pregunta como la suma ponderada de los porcentajes de respuesta sobre la cobertura percibida. El índice de percepción de cobertura en cada país se obtuvo como el promedio simple de las preguntas sobre cobertura en los grupos de población estudiados, tanto para antes como para después de la pandemia, según la ecuación:

$$I_{ij}=\frac{1}{5}(\sum_{p=1}^{5}\sum_{k=1}^{5}\mathrm{Porcentaje}\;{\mathrm{respuestas}}_{k}*\mathrm{Ponderador}))$$

donde, I es el índice de percepcion de cobertura, i es el país, j indica si la valoración es de antes o después de la pandemia, p es el grupo (embarazadas, recién nacidos, niños y niñas, adolescentes y mujeres), y k es un coeficiente de ponderación que depende de las posibles categorías para cada pregunta (1: muy baja/muy mala; 2: baja/mala; 3: media/aceptable; 4: alta/buena; 5: muy alta/muy mala).

**CUADRO 1.** Respuestas recibidas y analizadas por país

País	Respuestas recibidas (%)	Respuestas analizadas (%)
Argentina	33 (4,7)	33 (4,8)
Belice^a^	3 (0,4)	0
Bolivia	17 (2,4)	17 (2,4)
Brasil	36 (5,2)	36 (5,2)
Chile	215 (30,8)	215 (31,1)
Colombia	34 (4,9)	34 (4,9)
Costa Rica	19 (2,7)	19 (2,8)
Cuba	9 (1,3)	9 (1,3)
Ecuador	98 (14,1)	98 (14,2)
El Salvador	11 (1,6)	11 (1,6)
Guatemala	8 (1,1)	8 (1,2)
Guyana^a^	1 (0,1)	0
Haití^a^	2 (0,3)	0
Honduras	14 (2,0)	14 (2,0)
México	46 (6,6)	46 (6,7)
Nicaragua	10 (1,4)	10 (1,4)
Panamá	29 (4,2)	29 (4,2)
Paraguay	22 (3,2)	22 (3,2)
Perú	44 (6,3)	44 (6,4)
República Dominicana	12 (1,7)	12 (1,7)
Uruguay	23 (3,3)	23 (3,3)
Venezuela	11 (1,6)	11 (1,6)
Total	697 (100)	691 (100)

***Fuente:*** Elaboración propia.

a País no incluido en el análisis.

## RESULTADOS

Se obtuvieron respuestas de 697 informantes de 22 países; para el análisis solo se consideraron los países de los que se recibieron más de 3 respuestas, por lo que la muestra final de estudio quedó compuesta por 691 respuestas de 19 países (cuadro 1). El 75,1% de los informantes pertenecían al sector de la salud, con una experiencia laboral promedio de 21 años^2^.

### Percepción antes y durante la pandemia

Como se puede observar en el cuadro 2, la cobertura de servicios de salud para embarazadas, recién nacidos, niños y niñas, adolescentes y mujeres se percibía antes de la pandemia como “alta” o “muy alta” por el 52,9% los encuestados (24,3% y 28,6%, respectivamente), percepción que disminuyó al 39,5% al consultar sobre la calidad de esos servicios (25,7% y 13,8%, respectivamente).

A pesar de que la percepción del impacto sobre las transferencias monetarias (subsidios directos del gobierno), los servicios para las poblaciones vulnerables (migrantes, indígenas y afrodescendientes) y la provisión de beneficios sociales tuvieron porcentajes relativamente altos de respuestas registradas como no aplicables —cuando el encuestado consideraba que ninguna de las alternativas representaba su respuesta, no contaba con suficiente información para responderla o la pregunta no aplicaba a la realidad de su país—, la percepción mayoritaria de las respuestas sobre estos temas fue que se suspendieron de manera total o parcial. Esta percepción negativa se mantuvo en otros temas en los que el porcentaje de respuestas no aplicables fue bajo, como servicios de salud y servicios de salud específicos.

Sobre la evaluación de las respuestas institucionales durante la pandemia, el 42,8% fue buena o muy buena, similar a como se percibió la respuesta del sistema de salud (43,2%). Al analizar los resultados por tipo de institución, las gubernamentales (central, federal o subnacional, y local) tuvieron peor evaluación que las organizaciones no gubernamentales y el sector privado: mientras la percepción de una mala o muy mala respuesta por parte de las instituciones del sector público se ubicó entre el 25% y el 30% de las percepciones informadas, para el sector privado esa valoración negativa se redujo al 24,1%, para la academia al 21,2%, para las organizaciones no gubernamentales al 17,6% y para los organismos y organizaciones internacionales al 16,1%.

La percepción acerca de la respuesta del sistema de salud fue de mala o muy mala en 30,3% de las opiniones emitidas. De los servicios de salud del primer nivel de atención (promoción, prevención, cuidados, rehabilitación y urgencia), la atención de urgencia fue la mejor evaluada (el 48,9% la evaluó como buena o muy buena). Mientras, la mitad o más de las personas encuestadas percibió como buena o muy buena la respuesta de la atención en los niveles secundario (53,6%) y terciario (51,8%); menos del 25% la percibió como mala o muy mala.

### Proyección para después de la pandemia

Respecto a la situación después de la pandemia (cuadro 2), el 25,6% de las respuestas promedio de los países consideró que después de 3 meses los servicios aún mostrarían un retroceso considerable en comparación con la situación que existía antes de la pandemia, mientras el 46,9% opinó que la situación volvería al nivel anterior o a una situación mejor en ese lapso. Este pronóstico mejoró con vista a los 12 meses: el 58,7% opinó que los servicios volverían al nivel anterior a la pandemia o mejorarían. No obstante, se debe resaltar el hecho de que una proporción considerable de los encuestados dentro de cada país estimó que los servicios seguirían con una menor cobertura tanto a los 3 como a los 12 meses (53,1% y 41,3%, respectivamente). Estas consideraciones se confirman con los datos relevados durante la pandemia sobre la interrupción de servicios de salud tanto en el mundo como en la Región de las Américas (15–17).

Al analizar los datos por servicio, los que se identificaron con buena cobertura antes de la pandemia y menor interrupción durante ella —como los programas de inmunización y los de atención institucional del parto y posparto— se pronosticaron con una posible rápida recuperación. Del otro lado, la opinión sobre la evolución del acceso a métodos anticonceptivos, la salud mental, la prevención de infecciones de transmisión sexual, el control del niño sano y la medición del desarrollo infantil fue mayoritariamente en el sentido de que no se recuperarían las coberturas previas a la pandemia en los países en los siguientes 3 meses, pronóstico que mejoró al proyectarse a 12 meses.

En cuanto a la priorización dada a las problemáticas de las cinco poblaciones analizadas (embarazadas, recién nacidos, niños y niñas, adolescentes y mujeres) en la agenda pública, en promedio, 39,2% percibió que era baja o muy baja.

**CUADRO 2.** Percepción acerca de los servicios para embarazadas, recién nacidos, niñas y niños, adolescentes y mujeres en América Latina y el Caribe, según el período y el tema

Período/Tema	Categoría de respuesta, %^e^			N/A, %		
	Muy baja / muy mala	Baja / mala	Media / aceptable	Alta / buena	Muy alta / muy buena	
Antes de la pandemia^a^						
Cobertura de los servicios de salud	6,5	9,8	30,9	24,3	28,6	4,8
Calidad de los servicios de salud	4,6	15,3	40,5	25,7	13,8	3,2
Durante la pandemia^b^						
Beneficios sociales	7,7	43,9	31,4	14,5	2,5	13,8
Transferencias monetarias	6,9	33,0	40,1	11,7	8,3	39,5
Servicios de salud	9,3	43,7	30,4	14,5	2,1	5,2
Servicios de salud específicos	12,0	42,4	31,2	12,6	1,8	5,7
Servicios para poblaciones vulnerables^c^	12,3	47,3	23,2	15,3	2,0	22,6
Respuestas institucionales	7,2	18,9	31,0	30,1	12,7	11,1
Respuestas del sistema de salud	9,5	20,8	26,4	29,3	13,9	4,2
Priorización de temas	14,6	24,6	32,6	17,4	10,8	3,4
Proyección para después de la pandemia^d^						
Servicios de salud específicos (a los 3 meses)	25,6	27,5	33,6	13,3	6,4	
Servicios de salud específicos (a los 12 meses)	17,5	23,8	41,7	17,0	6,3	

***Fuente:*** Elaboración propia.

**Nota:** N/A: no aplicable; se refiere a las respuestas faltantes porque el encuestado consideraba que ninguna de las alternativas representaba su respuesta, no contaba con suficiente información para responder o no aplicaban a la realidad de su país.

a Significado de las categorías de respuesta: Muy baja / muy mala: menos de 30%; baja / mala: de 30 a 49%; media / aceptable: de 50 a 74%; alta / buena: de 75 a 89%; muy alta / muy buena: 90% o más.

b Significado de las categorías de respuesta: Muy baja / muy mala: suspendido; baja / mala: parcialmente reducidos; media / aceptable: igual que antes; alta / buena: adaptadas a las circunstancias; muy alta / muy buena: se crearon nuevos servicios.

c Migrantes, indígenas y afrodescendientes.

d Significado de las categorías de respuesta: Muy baja / muy mala: retroceso significativo; baja / mala: recuperación a un nivel menor; media / aceptable: igual que antes; alta / buena / muy alta/ muy buena: mejora.

e Porcentajes reportan el total de respuestas en las cinco alternativas, excluyendo la categoría N/A.

### Percepción sobre servicios de salud específicos

Las personas encuestadas percibían que, antes de la pandemia, los servicios tenían una alta o muy alta cobertura para embarazadas (60,7%), recién nacidos (67,6%), y niños y niñas (57,6%); esta percepción decreció al evaluar la cobertura de los servicios para adolescentes (35,6%) y mujeres (41,5%). En relación con la calidad, los servicios que se percibían con alta o muy alta calidad fueron los dirigidos a recién nacidos (52,6%), embarazadas (47,0%), niños y niñas (41,9%) y, en menor proporción, los dirigidos a mujeres (30,9%) y adolescentes (24,6%).

**FIGURA 1.**
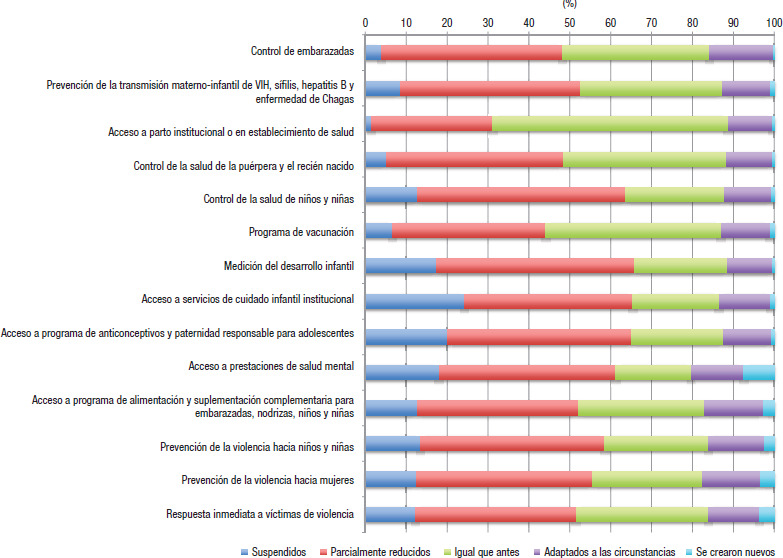
Percepción del impacto de la pandemia de COVID-19 sobre los servicios de salud en los países analizados de América Latina y el Caribe hasta septiembre del 2020

Los servicios para recién nacidos y embarazadas —como la atención institucional del parto y la atención del puerperio—, así como los programas de vacunación, muestran las mejores percepciones de cobertura durante la pandemia (figura 1). Por otro lado, acciones como el cuidado infantil, el acceso a los métodos anticonceptivos y los servicios de salud mental se percibieron como los más afectados. A pesar de una percepción de cobertura más baja, los servicios de salud mental se percibieron como los que más habían innovado —ya sea por adaptarse a las circunstancias o por crear servicios nuevos— para combatir los efectos de la pandemia.

### Resultados por país

Al analizar los servicios por país, se observó una alta heterogeneidad. La percepción de mayor cobertura y calidad fueron en Cuba, Costa Rica, Chile y Uruguay, mientras que las más bajas fueron en Guatemala, Honduras, Bolivia y Panamá.

**FIGURA 2.**
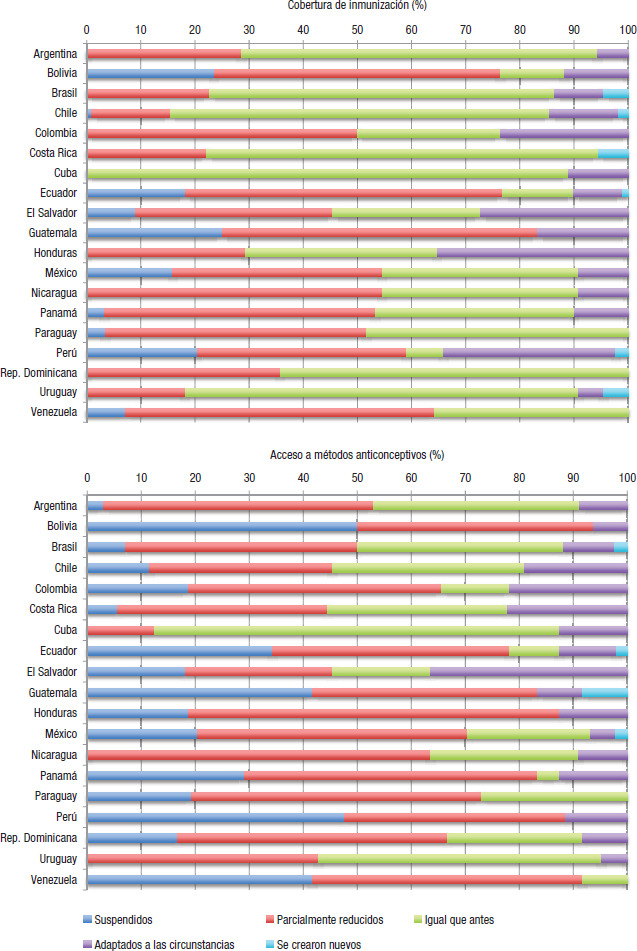
Percepción del impacto de la pandemia de COVID-19 sobre la cobertura de los servicios de inmunización y anticoncepción en los países analizados de América Latina y el Caribe hasta septiembre del 2020

Según las percepciones expresadas, la cobertura de inmunización, que era alta, se redujo en general durante la pandemia, con importantes diferencias entre países: mientras en Argentina, Brasil, Chile, Costa Rica, Cuba, República Dominicana y Uruguay más del 60% de los informantes percibieron que las coberturas de vacunación se mantuvieron durante la pandemia, en otros como Bolivia, Ecuador, Guatemala, México y Perú, el 15% o más de las respuestas informaban de la suspensión de estos servicios (figura 2).

El acceso a métodos anticonceptivos —que tenía una baja cobertura previa— presentó una mayor interrupción durante la pandemia. En promedio, más encuestados de cada país informaron que esta prestación se vio afectada durante la pandemia, aunque con grandes variaciones: mientras en Chile, Costa Rica, Cuba, El Salvador y Uruguay menos del 50% de los encuestados indicaron que los servicios se habían interrumpido de manera total o parcial, esta proporción se elevó a más del 80% en Bolivia, Guatemala, Honduras, Panamá, Perú y Venezuela.

**FIGURA 3.**
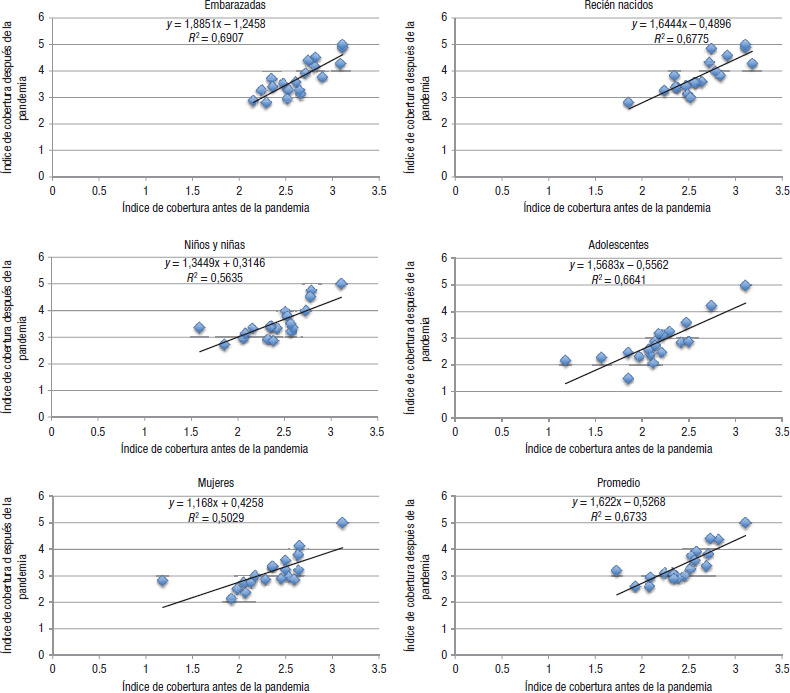
Percepción del impacto de la pandemia de COVID-19 en la cobertura de los servicios de salud en los países analizados de América Latina y el Caribe antes y después de declararse, por grupo de población

La relación entre la percepción sobre las coberturas antes y después de la pandemia en los cinco grupos de población estudiados, resultó directa y positiva; es decir, en los países donde una mayor proporción de los encuestados informó coberturas bajas antes de la pandemia, también una mayor fracción de los encuestados informó la interrupción total o parcial de servicios (figura 3).

### Áreas que se deben priorizar después de la pandemia

En línea con lo expuesto más arriba, el 40,6% de las respuestas indicaron que garantizar la cobertura y el acceso a los servicios de salud es el principal desafío político con vista al futuro, seguido del financiamiento de iniciativas para mujeres y NNA (23,5%) y la protección y promoción contra la violencia (13,1%) (cuadro 3).

***Fuente:*** Elaboración propia

No obstante, si bien “Garantizar el acceso a las prestaciones de salud” fue el principal desafío identificado en general, quedó relegado al segundo lugar en varios países, como en Brasil y Cuba (precedido por “Financiamiento a iniciativas para mujeres y NNA”), Costa Rica y Venezuela (precedido de “Protección y promoción contra la violencia”); y en Nicaragua compartió el primer lugar de prioridad con “Reasignación de roles y competencias a nivel subnacional o local”.

## DISCUSIÓN

Los resultados muestran que durante la pandemia los servicios de salud para embarazadas, recién nacidos, niñas, niños, adolescentes y mujeres han resultado “invisibilizados” en la agenda pública en una buena parte de los países de América Latina y el Caribe, a pesar de que, en promedio, la percepción sobre la mayor parte de los servicios de salud antes de la pandemia fue de una cobertura alta o muy alta y la percepción mayoritaria de su calidad era aceptable o buena. Estos resultados están alineados con lo informado por otros estudios respecto a las coberturas de salud antes de la pandemia (5, 6, 18, 19).

En los países analizados, una parte de los encuestados percibieron que los servicios de salud y los beneficios sociales se interrumpieron de forma total o parcial durante la pandemia. Los servicios en los que se percibió una menor interrupción fueron los dirigidos a recién nacidos, gestantes y programas de inmunización, mientras que los más afectados fueron los de cuidado infantil, acceso a métodos anticonceptivos, salud mental y medición del desarrollo infantil. Esto puede deberse a que se priorizaron los servicios con mayor impacto inmediato en la sobrevida de estos grupos de población para evitar su interrupción. Las interrupciones de servicios dirigidos a grupos vulnerables fueron de una magnitud similar a la observada en la población general de cada país, lo que no concuerda con resultados previos en los que se informaron inequidades mayores en estos grupos (4, 5).

Al analizar los efectos de la pandemia en los programas nacionales de inmunización y de acceso a métodos anticonceptivos, se observó que en los países donde la percepción de la cobertura inicial era baja, el efecto de la pandemia fue mayor. Esto podría confirmar aseveraciones anteriores en el sentido de que en los países con sistemas de salud más consolidados, la pandemia afectó en menor medida a la oferta regular de servicios de salud (5, 6).

***Fuente:*** Elaboración propia.

***Fuente:*** Elaboración propia

R^2^:estadístico de bondad de ajuste global que indica cuánto de la variación de y (índice de percepción después de la pandemia) es explicado por la variación de x (índice de percepción antes de la pandemia) en cada una de las ecuaciones de regresión lineal estimadas en los gráficos.

La interrupción del acceso a los métodos anticonceptivos en situaciones de emergencia se ha constatado en otras crisis sanitarias (20, 21) y durante la pandemia por SARS-CoV-2 (22). Respecto a los servicios de salud mental, si bien la cobertura antes de la pandemia era percibida como baja, se perciben como los más innovadores gracias a la implementación de soluciones para combatir sus efectos. Pese a ello, si se toma en cuenta la menor prioridad que tenían estos servicios (en cobertura y calidad) antes del inicio de la pandemia —incluidos otros servicios específicos, como los dirigidos a la salud de adolescentes, que han sido desatendidos en mayor medida durante la crisis sanitaria—, es necesario que los países creen y fortalezcan este tipo de servicios en el plazo más breve posible, para evitar efectos negativos de gran escala en el mediano y largo plazos (23).

La positiva evaluación de la respuesta de distintas instituciones o entidades podría explicarse por el reconocimiento de la pandemia como una urgencia para la que ningún país o institución estaba totalmente preparado.

Según las respuestas de los encuestados, en los países en los que la cobertura de servicios era alta antes de la pandemia se tendió a priorizar otros aspectos, distintos del acceso a los servicios para estas poblaciones, como la prevención de la violencia y el financiamiento de programas para mujeres y NNA. En los países con mayor acceso y calidad —capacidad de la salud pública, solidez de la atención primaria, capacidad hospitalaria e insumos suficientes— se estimó también que tenían una mejor preparación para afrontar la crisis (24), de ahí la importancia de fortalecer los sistemas de salud en todos sus niveles y para toda la población: la cobertura universal y de calidad de la atención contribuyen a la mejor preparación del sistema de salud y de las propias comunidades ante crisis sanitarias.

**CUADRO 3.** Valoración de cuál debe ser el área que más se necesita priorizar después de la pandemia para recién nacidos, niñas y niños y adolescentes (NNA), y mujeres en América Latina y el Caribe

Área	Valorada como la más importante, %
Garantía de cobertura y de acceso a los servicios de salud	40,6
Financiamiento para el desarrollo de acciones para mujeres y NNA	23,5
Protección y promoción contra la violencia	13,1
Reasignación de roles y competencias a nivel subnacional o local	10,8
Incremento de la cobertura para cuidados infantiles institucionales	6,4
Abogacía por los derechos de NNA	5,6

El presente estudio tiene algunas limitaciones que se deben tomar en cuenta al interpretar estos resultados. En primer lugar, dado que la encuesta fue aplicada mientras la pandemia estaba en desarrollo, parte de las percepciones sobre su efecto en los servicios de salud y las proyecciones de su recuperación a futuro podrían subestimar los efectos que la pandemia finalmente tuvo en la interrupción de los servicios en el 2020 y que continúa teniendo en el 2021. A esto se suma el impacto de la COVID-19 sobre las economías y las desigualdades dentro de la región, lo que puede acentuar algunos de los efectos negativos sobre los sistemas de salud (25–27). Asimismo, en los promedios por país se debe tomar en cuenta la variación en el número de respuestas por país y que, si bien el análisis incluyó un considerable número de países, en algunos de ellos no fue posible recabar toda la información necesaria sobre el efecto de la pandemia. Esa falta de información no es aleatoria y, al no contar con informantes de países que podrían tener coberturas iniciales más bajas, el impacto encontrado podría estar subestimado.

Pese a lo anterior, los hallazgos de este estudio permiten un acercamiento a la situación general en América Latina y el Caribe hasta septiembre del 2020, más allá de los informes oficiales emanados de las autoridades nacionales. Estos resultados están alineados con otros estudios de cobertura de servicios de salud antes de la pandemia y el impacto de ella (8, 9, 11). Para lograr una visión más integral de la evolución de la pandemia, se encuentra en proceso, en el marco del proyecto ISLAC, un segundo levantamiento durante el año 2021 que permitirá comparar la provisión de servicios a embarazadas, recién nacidos, niños y niñas, adolescentes y mujeres en América Latina y el Caribe en dos etapas de la pandemia.

Como conclusión, se constató el efecto negativo de la pandemia en los servicios de salud para los grupos de población analizados en América Latina y el Caribe. Aunque la pandemia ha golpeado a todos los países de la región y ha afectado a la provisión de servicios para todas las poblaciones analizadas, existen diferencias entre países y tipos de servicio; esta información puede ser muy útil para tomar decisiones más acertadas, en un contexto en el que esta es aún escasa.

Se requiere invertir en sistemas de información nacionales que permitan monitorear los distintos servicios e identificar las poblaciones que no se han priorizado. Adicionalmente, los países necesitan establecer estrategias para la mitigación de los efectos de la pandemia sobre los sistemas de salud y de recuperación de los servicios. Al hacerlo, deben tomar en consideración las necesidades y particularidades de cada grupo de población y reducir el impacto negativo sobre los más vulnerables en el corto, mediano y largo plazos. Dadas las particularidades de la pandemia y el contexto en que actualmente viven los países de América Latina y el Caribe, se requiere la implementación de soluciones innovadoras para enfrentar este importante reto.

## Declaración.

Las opiniones expresadas en este manuscrito son únicamente responsabilidad de los autores y no reflejan necesariamente los criterios ni la política de la *Revista Panamericana de Salud Pública / Pan American Journal of Public Health* y/o de la Organización Panamericana de la Salud.

## Footnotes

1 El cuestionario con todas las preguntas puede consultarse en el sitio web del proyecto ISLAC en https://www.islaccovid.org/
